# Genomic insights into the adaptation of *Acinetobacter johnsonii* RB2-047 to the heavy metal-contaminated subsurface mine environment

**DOI:** 10.1007/s10534-023-00555-0

**Published:** 2023-11-16

**Authors:** Ivana Timková, Lenka Maliničová, Lea Nosáľová, Mariana Kolesárová, Zuzana Lorková, Nikola Petrová, Peter Pristaš, Jana Kisková

**Affiliations:** 1 Department of Microbiology, Institute of Biology and Ecology, Faculty of Science, Pavol Jozef Šafárik University in Košice, Šrobárova 2, 04154 Košice, Slovakia; 2 Centre of Biosciences, Institute of Animal Physiology, Slovak Academy of Sciences, Šoltésovej 4-6, 04001 Košice, Slovakia

**Keywords:** *Acinetobacter johnsonii*, Antibiotics, Metals, Gold mine, Whole-genome sequencing, Efflux pumps

## Abstract

The subsurface mine environments characterized by high levels of toxic metals and low nutrient availability represent an extreme threat to bacterial persistence. In recent study, the genomic analysis of the *Acinetobacter johnsonii* strain RB2-047 isolated from the Rozália Gold Mine in Slovakia was performed. As expected, the studied isolate showed a high level of heavy metal tolerance (minimum inhibitory concentrations were 500 mg/L for copper and nickel, 1,500 mg/L for lead, and 250 mg/L for zinc). The RB2-047 strain also showed noticeable resistance to several antibiotics (ampicillin, kanamycin, chloramphenicol, tetracycline and ciprofloxacin). The genomic composition analysis demonstrated a low number of antibiotic and metal resistance coding genes, but a high occurrence of efflux transporter genes located on the bacterial chromosome. The experimental inhibition of efflux pumps resulted in decreased tolerance to Zn and Ni (but not to Cu and Pb) and to all antibiotics tested. In addition, the H33342 dye-accumulation assay confirmed the high efflux activity in the RB2-047 isolate. These findings showed the important role of efflux pumps in the adaptation of *Acinetobacter johsonii* strain RB2-047 to metal polluted mine environment as well as in development of multi-antibiotic resistance.

**Supplementary Information:**

The online version contains supplementary material available at 10.1007/s10534-023-00555-0.

## Introduction

In recent years, the investigation of microbial communities from extreme environments has led to many advances in molecular biology, medicine, as well as in biotechnology (Merino et al. 2019). Understanding of the mechanisms responsible for the ability of microorganisms to adapt to extreme environmental conditions is crucial from an evolutionary and ecological point of view. The structure of the microbial genome and the acquisition or loss of some genes through natural selection, genetic recombination, mutations or horizontal gene transfer play a significant role in their adaptation processes (Wani et al. 2022).

In this study, we focused on the identification of genetic determinants responsible for the ability of *Acinetobacter* sp. to adapt to the heavy metal-contaminated subsurface mine environment using whole-genome sequence analysis. Bacteria belonging to the *Acinetobacter* genus are Gram-negative, strictly aerobic, non-motile coccobacilli with lacking pigmentation. They inhabit diverse environments, including water, soil, food, and wastewater (Al Atrouni et al. 2016; Ghaima et al. 2018; Ekwanzala et al. 2020). In addition, they can be found as nosocomial pathogens and commensals in the clinical setting (Wong et al. 2017). Multiple antibiotic resistance of *Acinetobacter* spp. causes significant difficulties in treating infections. Firstly, multidrug resistance had been detected mainly in *A. baumannii* species, but later, it has also been reported in many other species (Kämpfer 2014).

As mentioned above, members of the genus *Acinetobacter* have been isolated from various environments, including areas contaminated with heavy metals (Ghaima et al. 2018; Ekwanzala et al. 2020). Heavy metal pollution of soil and water is a global environmental problem as heavy metals are toxic to living organisms in certain concentrations, not degradable and difficult to remove from the contaminated environment (Sodhi et al. 2022). Heavy metals are stressors that could activate various protective and/or adaptive responses in bacteria, e.g., active efflux of the substance out of the cell, reduced influx of the target substance into the cell, enzymatic modification of the target molecule or its target site in the cell (Poole 2012; Kämpfer 2014).

Several genetic determinants involved in heavy-metal tolerance have been identified in *Acinetobacter* spp. with efflux pumps appearing to be the most prevalent adaptive mechanism to heavy metals, e.g. *cop* operon consisting of genes involving in copper ion efflux from the cell, *mer* operon involved in the transport of mercury ions out of the cell, *chr *operon with *chrA* and *chrB* genes encoding proteins involved in chromium tolerance, *czc* operon consisting of genes encoding CzcABCD proteins, which ensure efflux of cobalt, zinc and cadmium cations out of the cell or *ars* operon consisting of genes associated with the reduction of arsenate to arsenite and its efflux from the bacterial cell (Mindlin et al. 2016; Marwa et al. 2019; Bazzi et al. 2020; Petrova et al. 2023).

Interestingly, many studies have been documented that heavy-metal presence in the environment also leads to the development of bacterial multiple antibiotic resistance (reviewed in Squadrone 2020; Vats et al. 2022, Sodhi et al. 2023). Metal-induced co-selection for antibiotic resistance poses a risk of the expansion of the soil bacterial resistome, even under conditions of isolation of the bacterial community from antibiotic residues (Timková et al. 2020). The physical co-location and linkage of genes encoding microbial resistance to metals and antibiotics on the same genetic element (chromosome, integrons, transposons, or plasmids) could result in the phenomenon called cross-resistance, which occurs when only one gene encodes resistance to both, metals and antibiotics (Zhao et al. 2019). Despite the high prevalence of *Acinetobacter* spp. in heavy-metal contaminated environments, there are few studies addressing genetic determinants of antibiotic resistance induced by the presence of heavy metals in this genus. However, multiple resistance to heavy metals seems to be often linked with the presence of beta-lactamase genes (Furlan et al. 2019; Jia et al. 2021; Petrova et al. 2023). From this point of view, it was also interesting to exam the antibiotic susceptibility and its genetic determinants of the *Acinetobacter* isolate obtained from an isolated mine environment.

## Materials and methods

### Origin of the RB2-047 isolate

The RB2-047 isolate was obtained from the gold-bearing ore bacterial community of the Rozália Gold Mine in Hodruša-Hámre village (Slovakia) according to our previous study (Timková et al. 2020). Mining activities in the region were mainly focused on gold and silver ore mining and processing in the past, while today mining is focused mainly on gold, and less on copper, silver and lead (Sejkora et al. 2015; Chovan et al. 2016). The ore sample was collected directly in the mine from the depth of about 600 m below the surface (14th level of the Rozália mine) 3 days after the blasting and initiation of mining activity. The collected ore material was characterized by high concentrations of metals such as Zn (1,455 mg/kg), Fe (35,902 mg/kg), Mn (21,967 mg/kg), Cd (9 mg/kg) and Pb (653 mg/kg) (Timková et al. 2020). The primary identification of the isolate was performed using Matrix-Assisted Laser Desorption/Ionization Time-Of-Flight Mass Spectrometry (MALDI-TOF MS) and it was identified as *Acinetobacter johnsonii* with the score value of 2.188 according to our previous study (Nosáľová et al. 2021).

### Metal and antibiotic tolerance testing

Metal and antibiotic tolerance of the RB2-047 isolate was examined by the agar-dilution method and the minimum inhibitory concentration (MIC) was determined as the lowest metal or antibiotic concentration resulting in non-visible growth of the isolate (Schumacher et al. 2018; Timková et al. 2020). The RB2-047 isolate was inoculated onto the Mueller-Hinton agar (Merck KGaA, Germany) supplemented with one of the selected metals or antibiotics to final concentration, and cultivated at the laboratory temperature (~ 25 °C) for 48 h. Heavy metals were added to the culture medium in the form of ZnCl_2_, NiCl_2_·6H_2_O, CuCl_2_·2H_2_O or Pb(C_2_H_3_O_2_)_2_·3H_2_O solutions (Centralchem, Slovakia) to final concentrations of the ZnCl_2_, NiCl_2_ and CuCl_2_ ranged from 2 to 1,000 mg/L, concentrations of Pb(C_2_H_3_O_2_)_2_ ranged from 2 to 2,000 mg/L. The final concentrations of ampicillin ranged from 0.25 to 400 mg/L, concentrations of kanamycin, chloramphenicol, tetracycline and ciprofloxacin ranged from 0.25 to 10 mg/L in the culture medium (SERVA, Germany).

In addition, the agar-dilution method was used to examinate the effect of efflux pump inhibitor cyanide 3-chlorophenylhydrazone (CCCP) (Sigma-Aldrich, Germany) on heavy-metal and antibiotic tolerance of the RB2-047 isolate. Bacterial cells were inoculated onto the Mueller-Hinton agar supplemented with heavy metals or antibiotics using the same concentrations as described above and CCCP with a final concentration of 50 µM CCCP. The growth of the isolate was compared against control without CCCP addition.

### Measurement of the efflux pump activity using H33342 dye-accumulation assay

The efflux pump activity in the RB2-047 isolate was investigated using the Hoechst 33,342 (H33342) dye-accumulation assay and efflux pump inhibitor CCCP (Choi et al. 2017). *Escherichia coli* strain 1–22 carrying the *tetA* gene encoding the efflux pump responsible for tetracycline resistance was used as a positive control.

Five microliters of Luria-Bertani (LB) broth (Merck, Germany) were inoculated by 1 µL of bacterial culture and cultivated at 25 °C (RB2-047 isolate) or at 37 °C (*E. coli* 1–22) for 18 h. Subsequently, bacterial culture was diluted with LB broth to a value of 0.5 McFarland turbidity standard (McFarland densitometer, Biosan, Latvia). Bacterial culture of 176 µL was transferred to the wells of a 96-well microtiter plate with or without 50 µM efflux pump inhibitor CCCP and then 2.5 µM H33342 was added to each well. Each bacterial culture, the RB2-047 strain as well as control, was tested in triplicates in the presence and absence of CCCP. Fluorescence was measured at 37 °C every 10 min for 2 h using the Fluorescence microplate reader (BMG LABTECH, Germany). Then, the H33342 accumulation ratio (HAR) was calculated as the average amount of H33342 accumulated in the presence of CCCP (HAC) divided by H33342 accumulated in the absence of CCCP (HA).

### DNA extraction

The total genomic DNA of the RB2-047 isolate was extracted from the overnight culture cultivated in Luria Bertani broth (Merck KgaA, Germany) using the GenElute Bacterial Genomic DNA Kit (Sigma-Aldrich, USA). The quality of the extracted DNA was inspected by electrophoresis in a 1% agarose gel stained with ethidium bromide (0.5 µg/L) and visualized under UV light using the GEL LOGIC 212 PRO detection system (Carestream, USA). DNA concentration and purity were measured using NanoDrop 2000c Spectrophotometer (Thermo Scientific, USA).

### The 16S rRNA gene sequence analysis

The 16S rRNA gene was amplified according to the protocol of Nosáľová et al. (2021). PCR amplicons were sequenced in both directions using the Sanger sequencing method by Eurofins Genomics (Köln, Germany). The sequences obtained were assembled using the CAP3 tool (Huang and Madan 1999) and analyzed using the BLASTN search tool against the GenBank 16S rRNA sequence database (Altschul et al. 1990).

The phylogenetic analysis was performed using the MEGAX v10.2.4 software (Kumar et al. 2018). The 16S rDNA sequence was aligned with other 16S rRNA gene sequences of representatives of the *Acinetobacter* genus obtained from the GenBank database using the ClustalW tool. The phylogenetic tree was constructed using the Neighbor-Joining algorithm with 1,000 bootstrap replications. The evolutionary distances were calculated using Kimura 2-parameter model (Kimura 1980).

### The whole-genome sequence analysis

Whole-genome sequencing of the RB2-047 isolate was performed using the Illumina HiSeq 2000 technology by paired-end strategy (2 × 150 bp) in NovaSeq 6000 mode using S4 PE150 XP kit (Eurofins Genomics Europe Sequencing GmbH, Köln, Germany).

The raw reads were further processed by tools implemented in the Unipro UGENE v35.0 cross-platform bioinformatic software (Okonechnikov et al. 2012; Golosova et al. 2014). The quality of sequences was evaluated by the FastQC v0.11.9 tool, followed by sequence trimming using the Trimmomatic v0.39 tool with the minimum average quality threshold of 20. Filtered reads were *de novo* assembled using the SPAdes v3.12.0 tool and contigs shorter than 200 bp were excluded from further analyses.

The draft genome sequence was annotated by the RAST annotation server (Aziz et al. 2008; Overbeek et al. 2014; Brettin et al. 2015) and the results were checked using the BLASTN search tool (Altschul et al. 1990). The complete chromosome and plasmid sequences of the RB2-047 isolate were deposited in the GenBank database under the accession number JADDYQ000000000.

Bacterial genomic relatedness was examined by the average nucleotide identity (ANI) and in silico DNA-DNA hybridization (DDH) analysis. The ANI value was calculated to compare the genome sequences of the RB2-047 isolate to reference sequences of *Acinetobacter* spp. using the Kostas Lab average nucleotide identity online calculator with the cut-off value of 95% indicating the same species (http://enve-omics.ce.gatech.edu/ani/) (Goris et al. 2007; Rodriguez-R and Konstantinidis 2014). In silico DNA–DNA hybridization analysis was performed using the Type (Strain) Genome Server (TYGS) with the recommended species cut-off value of 70% (Meier-Kolthoff and Göker 2019). TYGS was also used to examine the genome BLAST distance phylogeny (GBDP) to determine the phylogenetic position of the RB2-047 isolate within the genus *Acinetobacter*.

### Identification of metal tolerance and antibiotic resistance determinants

Genetic determinants of metal tolerance and antibiotic resistance of the RB2-047 isolate were predicted using RAST annotation server (Aziz et al. 2008; Overbeek et al. 2014; Brettin et al. 2015). The presence of antibiotic resistance genes were investigated using the ResFinder 4.1 tool (Zankari et al. 2017; Bortolaia et al. 2020) and Comprehensive Antibiotic Resistance Database (CARD) (Alock et al. 2023). All results were subsequently verified using the BLASTN search tool (Altschul et al. 1990).

In addition, in silico comparative genomic analysis of the RB2-047 isolate was performed using other *A. johnsonii* genome sequences obtained from the GenBank database, while strains were isolated from different environments. All *A. johnsonii* genomes were analyzed using RAST annotation server, ResFinder 4.1 tool and CARD.

### The plasmid sequence analysis

The server mlplasmids v2.1.0 was used to identify plasmid related sequences using *Acinetobacter baumanii* as a model (https://sarredondo.shinyapps.io/mlplasmids/) (Arredondo-Alonso et al. 2018). Plasmid suspected sequences were examined using the ORF finder tool (https://www.ncbi.nlm.nih.gov/orffinder/), and open reading frames identified were analyzed using the BLASTP search tool (Altschul et al. 1990). In parallel, the plasmid suspected sequences were annotated using the Bakta tool (Schwenger et al. 2021).

The presence of toxin/antitoxin (TA) genes were determined using TADB 2.0, an online bacterial type II TA loci prediction tool (Xie et al. 2018). The list of toxins/antitoxin genes was completed based on the results of the TADB 2.0 annotation process. All TA genes were subsequently verified by the BLASTX search tool using NCBI Uni-ProtKB and Reference protein databases (Altschul et al. 1990).

Plasmid maps were constructed using ApE—A plasmid Editor v3.1.3 (Davis and Jorgensen 2022).

### Statistical analysis of the data

Results were analyzed using statistical tools implemented in LibreOffice Calc v7.3.6.2. The accumulation of H33342 dye was evaluated by one-way ANOVA and a *p*-value of 0.05 was used as the cut-off for statistical significance. Difference between the H33342 dye accumulation in the absence/presence of efflux pump inhibitor CCCP was assessed by Student’s *t*-test and a *p*-value of 0.05 was used as the cut-off for statistical significance.

## Results

### Metal and antibiotic tolerance testing

**Fig. 1:**
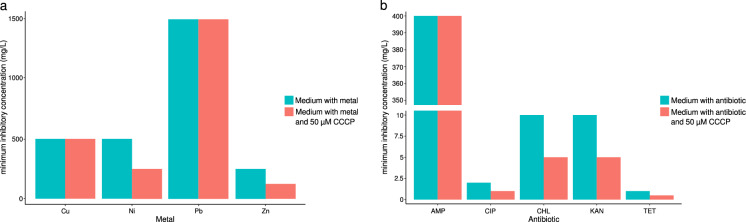
Influence of efflux pump inhibitor CCCP on minimum inhibitory concentration of selected metals (**a**) and antibiotics (**b**) in the RB2-047 isolate. *CCCP* cyanide 3-chlorophenylhydrazone, *AMP* ampicillin, *CIP* ciprofloxacin, *CHL* chloramphenicol, *KAN* kanamycin, *TET* tetracycline

The RB2-047 isolate showed the highest degree of tolerance towards lead (1,500 mg/L), and the lowest tolerance to zinc (250 mg/L). The addition of 50 µM CCCP to the culture medium led to observable decrease of MIC value for Ni and Zn but not for Pb and Cu in the agar-dilution testing of the efflux pump activity (Fig. 1a).

The RB2-047 isolate showed the highest resistance to ampicillin (MIC value of 400 mg/L) and the highest sensitivity to tetracycline (MIC value of 1 mg/L). The presence of CCCP significantly decreased tolerance of the RB2-047 isolate to kanamycin, chloramphenicol, tetracycline and ciprofloxacin (MIC reached half of the previous values) but tolerance to ampicillin was not affected by the inhibitor (Fig. 1b).

### Measurement of the efflux pump activity using H33342 dye-accumulation assay

**Fig. 2:**
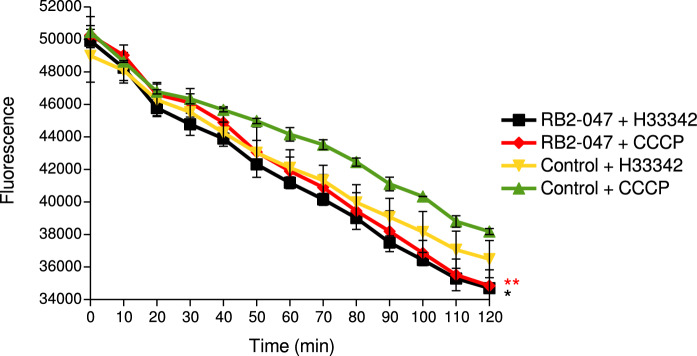
Measurement of the Hoechst H33342 dye accumulation in the RB2-047 isolate and control in the presence and absence of efflux pump inhibitor CCCP. CCCP cyanide 3-chlorophenylhydrazone; *significant reduction of Hoechst H33342 dye accumulation over time (*p* < 0.05, one-way ANOVA); **significant reduction of efflux pump activity in the presence of CCCP compared to the absence of inhibitor (*p* < 0.05, Student’s *t*-test)

The highest level of H33342 accumulation was recorded at the beginning of the measurement, which decreased significantly over time (*p* < 0.05, one-way ANOVA). This finding could point to the activation of efflux pumps. The dye accumulation showed highly similar trend in the presence of CCCP, but the fluorescence values were significantly higher (*p* < 0.05, Student’s *t*-test), i.e., the activity of efflux pumps was inhibited and the efflux of H33342 from bacterial cells was reduced (Fig. 2).

**Fig. 3:**
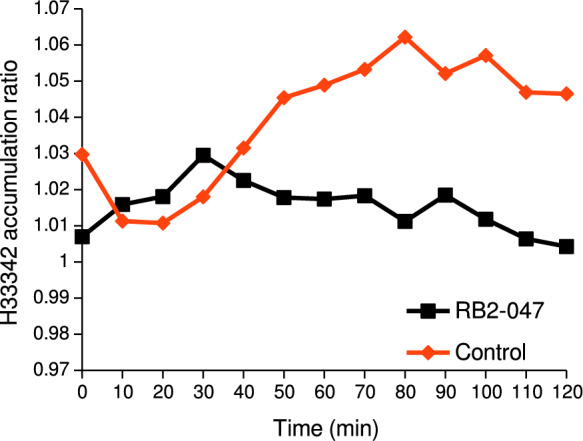
Efflux pump activity in the RB2-047 isolate assessed using the H33342 accumulation ratio

The difference in H33342 accumulation in the presence and absence of CCCP was the highest after 30 min. Then, after 50 min of measurement, the HAR decreased and remained approximately at the same level until decline after 90 min (Fig. 3).

### The 16S rRNA gene sequence analysis

The 16S rRNA gene sequence (1,418 bp) of the RB2-047 isolate showed a 99.51% similarity to the sequence of *A. johnsonii* strain ATCC 17,909 deposited in the GenBank database (MK184297).

**Fig. 4:**
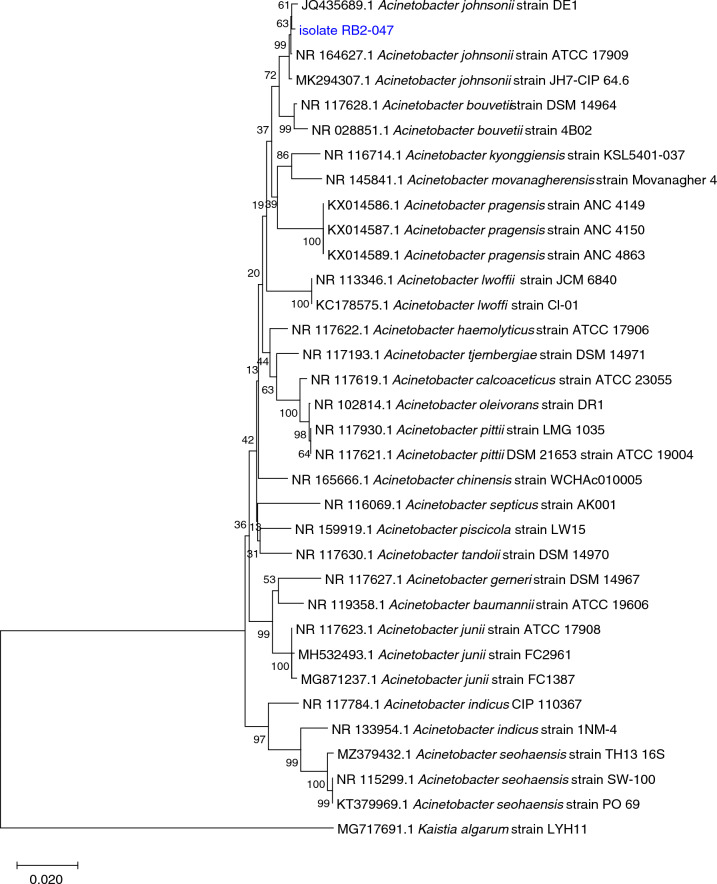
Phylogenetic relatedness of the RB2-047 isolates within *Acinetobacter* spp. based on 16S rRNA gene sequences. Phylogenetic tree was constructed using the Neighbour-Joining method and the evolution distances were calculated using the Kimura 2-parameter model. Bootstrap values ≥ 50% based on 1000 replications are shown at branch nodes. The 16S rRNA gene sequence of *Kaistia algarum* LYH11 (MG717691.1) was used as an outgroup

The phylogenetic analysis based on the 16S rRNA gene sequences confirmed the closest relationship of the RB2-047 isolate to the *A. johnsonii* group (Fig. 4).

### The whole-genome sequencing analysis

A total of 7,903,000 raw reads were obtained using whole-genome sequencing giving 700x coverage of the RB2-047 genome. These reads were assembled into the draft genome of 3,378,761 bp consisting of 518 contigs with N50 of 38,820 bp, a maximum contig length of 214,542 bp and the G + C content of 41.8%. In addition, two sequences were identified as complete circular plasmids.

The ANI value between the RB2-047 genome and closely related *A. johnsonii* DSMZ genome (NZ_BBTB00000000.1) was calculated as 95.86%, which is slightly above the threshold cut-off value of 95% for the same species classification according to Goris et al. (2007).

**Fig. 5:**
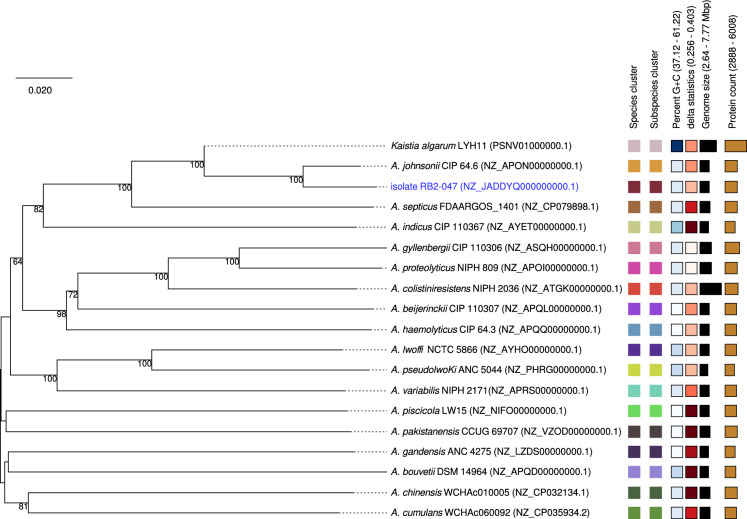
Genome BLAST distance phylogeny (GBDP) showing the relatedness of the RB2-047 isolate to related *Acinetobacter* species. The GBDP phylogram was generated using the Type (Strain) Genome Server. The numbers shown at branch nodes are GBDP pseudo-bootstrap values ≥ 50% based on 1000 replications. Leaf labels with different colors indicate species and subspecies clusters; the color range (from light to dark) increases based on genomic G + C content and delta statistics values (Meier-Kolthoff and Göker 2019)

In silico DDH value between the RB2-047 isolate and its closest relative (*A. johnsonii* CIP 64.6, GenBank accession number NZ_APON00000000.1) reached the threshold value of 70% for the same species classification. However, the phylogenetic analysis based on the in silico DDH clearly confirmed the close relationship of the RB2-047 isolate with *A. johnsonii* species (Fig. 5).

### Identification of metal and antibiotic resistance determinants

Genome annotation revealed the presence of various genes associated with antibiotic and/or metal tolerance of the RB2-047 isolate. All these genetic determinants were located on the bacterial chromosome (Table 1).

**Table 1:** Metal tolerance and antibiotic resistance genes identified using whole-genome sequencing in the RB2-047 isolate

Mechanism	Gene	Function	Size (bp)	Amino acid sequence similarity GenBank Accession No. % of similarity
Efflux transporters	*bcr*	Bcr/ClfA family efflux transporter	1,203	WP_151836224.1 100
	*pATP*	heavy metal translocating P-type ATPase	2958	WP_205666914.1 98.38
	*tolC*	Outer membrane protein of multidrug efflux pumps	1,062	WP_114392930.1 100
	*corC*	magnesium/cobalt efflux transporter	840	CAB1219066.1 95
	*ydhE/norM*	MATE family efflux transporter	1,368	WP_125280852.1 99.78
	*chrA*	chromate efflux transporter	1,359	WP_094149097.1 99.12
	*chrB*	chromate efflux transporter	921	WP_005399446.1 100
	*czcA*	cobalt/zinc/cadmium efflux transporter	3147	WP_004979700.1 98.95
	*czcB*	cobalt/zinc/cadmium efflux transporter	1236	WP_004979701.1 98.54
	*czcC*	cobalt/zinc/cadmium efflux transporter	1323	WP_004979702.1 96.14
	*czcD*	cobalt/zinc/cadmium efflux transporter	1281	WP_089606599.1 98.36
	*macA*	macrolide-efflux transporter	1,335	WP_114837681.1 99.32
	*qacJ*	small multidrug resistance efflux transporter	366	ENU38662.1 99.17
Metal resistance	*copB*	copper resistance protein	921	WP_205667080.1 99.03
	*copC*	copper resistance protein	381	WP_057061708.1 100
	*copD*	copper resistance protein	882	WP_151723786.1 97.89
Regulators	*merR*	mercury-responsive transcriptional regulator	639	WP_126037285.1 100
	*cusS*	copper-responsive transcriptional regulator	1,401	WP_195728335.1 97.64
	*cusR*	copper-responsive transcriptional regulator	681	WP_126034993.1 99
	*cadR*	Cd(II)/Pb(II)-responsive transcriptional regulator	393	WP_005401421.1 99
Antibiotic resistance	*bla*_*OXA−211*_	beta-lactamase OXA-211	825	WP_063861677.1 100

Most genes encode efflux transporter proteins belonging to the ATP-binding cassette (ABC) transporters. We identified genes encoding metal transport proteins such as *corC* or *chrA/B*, also multidrug transport protein genes were detected (e.g., brc, *ydhE/norM, macA*).

Copper tolerance is mediated by several cooper resistance genes (*copBCD*) and transcriptional regulators (*cusS*, *cusR*). In addition, we found genes of *czc* operon which ensure efflux of cobalt, zinc and cadmium cations out of the cell and genes encoding regulator transcription factors associated with the tolerance to mercury (*merR*) and cadmium and lead (*cadR).*

Only one antibiotic resistance gene encoding beta-lactamase OXA-211 with carbapenemase activity was found in the RB2-047 genome.

### In silico comparative genomic analysis of the RB2-047 isolate

Five *A. johnsonii* genome sequences obtained from the GenBank database were used for in silico comparative genomic analysis of the RB2-047 isolate. These isolates were found in different environments. The genome of the RB2-047 isolate showed the highest number of protein-coding genes within the *Acinetobacter* collection, while the GC content or the number of tRNA genes were similar among the isolates. The number of antibiotic resistance genes was the highest in the clinical isolate XBB1 (10), followed by the Acsw19 isolate from sewage (5). Only one antibiotic resistance gene (*bla*_*OXA−211*_ or *bla*_*OXA−373*_) was found in other isolates (Table 2).

**Table 2:** Comparison of genome features and genetic determinants of heavy-metal tolerance and antibiotic resistance in various *Acinetobacter johnsonii* strains

Category/isolate	RB2-047	XBB1	M19	IC001	Acsw19	LXL C1
GenBank Accession No.	JADDYQ000000000	NZ_CP010350	NZ_CP037424	NZ_CP022298	NZ_CP043307	NZ_CP031011
Source	Mine	Clinical environment	Water	Water	Sewage	Soil
Genome size (Mbp)	3.39	3.51	3.75	3.61	3.43	3.40
% GC	41.8	41.4	41.4	41.5	41.8	41.3
Plasmid No.	≥ 3	8	1	4	3	0
Protein-coding genes No.	3,518	3,139	3,450	3,203	3,049	2,975
tRNAs No.	84	83	88	88	88	87
Copper tolerance and homeostasis	*copBCD, cusRS*	*copBCD, copZ, cusRS*	*copBCD, copZ, cusRS*	*copBCD, copG, copZ, cusRS*	*copB*	*copBCD, cusRS*
Co–Zn-Cd resistance	*czcABCD*	*czrR*,	*czcCD, czrR*	*czcCD, czsB, czrR*	*czcD*	*czcCD, czrR*
Non-specific efflux pumps	MATE, *corC, tolC, bcr, macA*,	MATE, *corC, tolC, bcr, macA*,	MATE, *macA*	MATE, *macA*	MATE, *macA*	MATE, *macA*
Chromium resistance	*chrAB*	*chrAB*	*chrAB*	*chrAB*	*chrA*	*chrAB*
Mercury resistance	*merR*	*merR*,				
Aminoglycoside resistance		*aac(3)(6′), aph(3″)-Ib*, *aph(6)-Id aph(3′)-VIa*			*5aph(3″)-Ib, aph(6)-Id*	
Beta-lactam resistance	*bla*_*OXA−211*_	*bla*_*OXA−58*_, *bla*_*PER−1*_	*bla*_*OXA−211*_	*bla*_*OXA−333*_	*bla*_*OXA−373*_, *bla*_*NDM−1*_	*bla*_*OXA−373*_
Macrolide resistance		*mph(E), msr(E)*				
Tetracycline resistance		*tet(Y)*				
Phenicol resistance					*floR*	

Copper resistance gene *copB* or copper resistance gene regulatory elements *cusRS* were present in all compared isolates except for *A. johnsonii* Acsw19. Similarly, copper or chromium resistance genes (*copBCD* or *chrAB*) were present in all compared bacteria except for *A. johnsonii* Acsw19 (Table 2).

We noticed the presence of specific beta-lactam resistance genes in all compared isolates. Only a single *bla*_*OXA−211*_ gene was found in the RB2-047 genome; as well as in the *A. johnsonii* M19 genome.

The RB2-047 isolate showed the presence of various efflux pump genetic determinants responsible for multi-resistance against a wide range of compounds. Similarly high number of efflux pump determinants was found in the clinical isolate *A. johnsonii* XBB1, also a significantly higher number of antibiotic resistance genes (e.g., *aph(6)-Id, bla*_*PER−1*_, *bla*_*OXA−58*_, *tet(Y)*) was confirmed.

### The plasmid analysis

The genome sequences of the RB2-047 isolate included two complete circular plasmids with a size of 4.9 kb (RB2-047-1) and 2.3 kb (RB2-047-2) (Online Resource 1). Another uncomplete plasmid contig (NODE_73) was identified using the mlplasmid server v2.1.0. A detailed annotation of plasmid’s sequences is shown in Table 3.

**Table 3:** The genetic analysis of the RB2-047 isolate plasmid related sequences

Plasmid name	Size (bp)	GC content (%)	ORFs No.	Replication initiation protein	Predicted maintenance and transfer genetic modules	Other genes
NODE_73	> 11,360	36.6	11	RepM	Type II toxin-antitoxin system RelE/ParE family toxin, Antitoxin HigA1, plasmid mobilization relaxosome protein MobC, relaxase/mobilization nuclease domain protein	MvaI/BcnI family restriction modification system, metal/formaldehyde-sensitive transcriptional repressor (RcnR-like protein)
RB2-047-1	4940	38.2	24	pfam03090 replicase superfamily replication initiation protein	Type II toxin-antitoxin system RelE/ParE family toxin, plasmid mobilization relaxosome protein MobC, relaxase/mobilization nuclease domain protein	
RB2-047-2	2,267	37.8	11	Rep63		

No genetic determinants of metal tolerance or antibiotic resistance were detected in analyzed plasmids. All plasmid-like sequences showed GC content lower than rest of the RB2-047 genome and encoded genes necessary for plasmid replication and maintenance only.

## Discussion

The present study follows our previous work of Timkova et al. (2020), which demonstrated a positive correlation between increased ampicillin-chloramphenicol and nickel-copper tolerance as well as a linkage between increased tetracycline-kanamycin and zinc-lead tolerance in the bacterial community from the mine subsurface environment (Rozália Gold Mine in Hodruša-Hámre village, Slovakia). The RB2-047 strain isolated from the same environment was identified as *Acinetobacter johnsonii* by polyphasic approach—a combination of MALDI-TOF MS (Nosáľová et al. 2021) as well as 16S rRNA gene and whole-genome sequence analysis (this study). It showed multiple antibiotic resistance (to ampicillin, kanamycin, chloramphenicol, tetracycline and ciprofloxacin) and high tolerance to several heavy metals (Zn, Ni, Cu and Pb). The RB2-047 isolate showed significantly higher MIC values of selected metals compared to the *A. lwoffii* strain ZS207 isolated from former gold and arsenic mine, where MIC observed reached a value of 114 mg/L for copper, 106 mg/L for Ni and 92 mg/L for Zn (Walter et al. 2020).

Most of the genes associated with heavy-metal resistance in the BR2-047 isolate encoded efflux transporters. The high efflux pump activity was also confirmed by the H33342 accumulation test in the presence/absence of the efflux pump inhibitor CCCP. In addition, lower MIC for Ni and Zn in the presence of the inhibitor in the agar-dilution test confirmed the important role of efflux pumps in the adaptation of bacterium to the presence of these heavy metals in the environment. On the other hand, the efflux activity appears to be less efficient in the development of Cu and Pb tolerance or it is possible that the activity of the efflux pumps transporting Cu and Pb out of the cell was not affected by CCCP. The high Pb tolerance of the RB2-047 isolate could be provided by non-specific efflux pumps (e.g., metal transporting ATPase) or other mechanisms, as we did not detect any genes in the RB2-047 genome that could participate in the development of Pb tolerance (e.g., *pbr* operon). Very similar genetic determinants were also found in the *Acinetobacter* species isolated from a brown mud created during aluminum production near Ziar nad Hronom (Slovakia), and the isolate showed a similarly high tolerance to the same metals (Petrová et al. 2023). Similar findings reported Furlan et al. (2019) in multi-drug resistant *Acinetobacter seifertii* obtained from soil of a corn crop field. According to in silico comparative genomic analysis, *Acinetobacter johnsonii* isolates found in environments with high metal (RB2-047 and XBB1) showed similar numbers and types of efflux pump genes. This fact points to the importance of the efflux mechanism in the adaptation of bacteria to extreme conditions (this study).

Generally, efflux activity is one of the fastest and most effective mechanism in the bacterial repertoire of stress responses and many of them have also been found among *Acinetobacter* spp. (Hassan et al. 2013; Alcalde-Rico et al. 2016; Kornelsen and Kumar 2021). For the first time, the genes encoding proteins potentially involved in the efflux, transport or reduction of antimicrobials, as well as heavy metals, were identified in the genome of *A. johnsonii* originated from creosote-polluted groundwater in Fredensborg (Denmark) (Kaas et al. 2017). Multidrug resistant *A. johnsonii* strains have been isolated from a variety of environments, e.g., from the Ba River and the Tiaozi River in China (Zhao et al. 2014; Jia et al. 2021), or from antimony mine tailings in Nandan County (China) (Gu et al. 2020).

Multi-antibiotic resistance of *Acinetobacter* spp. has been recorded mainly in clinical setting but have also been well documented in environmental isolates (including *A. johnsonii*) (Eze et al. 2022; Hubeny et al. 2022; Mapipa et al. 2022; Kisková et al. 2023). The natural resistance to various antibiotic compounds and the ability to develop a new antibiotic resistance under selection pressure is the most remarkable characteristic of *Acinetobacter* species (Kittinger et al. 2018; Furlan et al. 2019; Din et al. 2021). Our results showed that efflux pumps also play an important role in the development of multi-antibiotic resistance in environmental isolates of *Acinetobacter johnsonii* species. The effect of the CCCP inhibitor on the efflux activity was less apparent in the presence of ampicillin in the culture medium. This finding suggests that other mechanisms are also significantly involved in the development of ampicillin resistance, e.g., the presence of beta-lactamases, in the RB2-047 isolate.

Analysis of the RB2-047 genome revealed the presence of only one chromosomally located antibiotic resistance gene *bla*_*OXA-211*_ belonging to the group of beta-lactamase genes. This gene appears to be found only in the genus *Acinetobacter* according to BLASTN analysis. Recently, more than 210 beta-lactamases (including several types of oxacillinases, OXA enzymes) have been identified in the genus *Acinetobacter* (Zhao and Hu 2012; Hubeny et al. 2022; Nogbou et al. 2022). Also, a variety of beta-lactamase genes were identified in *A. johnsonii* (Espinal and Roca 2011; Montaña et al. 2016; Baraka et al. 2021). Zhao and Hu (2012) demonstrated that the spread of *Acinetobacter* oxacillinases into other species is more limited than other beta-lactamase genes and they enables *Acinetobacter* spp. to adapt easily to newly developed beta-lactam antibiotics. The identification of beta-lactamase genes and other antibiotic resistance determinants among *Acinetobacter* species demonstrates their potential to acquire and stably maintain resistance determinants in their genomes (Montaña et al. 2016). Beta-lactamase genes appear to be linked to a similar profile of heavy-metal tolerance determinants as shown by the RB2-047 isolate (Furlan et al. 2019; Petrová et al. 2023). In silico comparative genomic analysis confirmed these findings also in other environmental *Acinetobacter johnsonii* species (this study).

Environmental isolates M19, IC001, LXL C1 and RB2-047 showed a significantly lower number of antibiotic resistance genes compared to the clinical isolate XBB1 or the Acw19 isolate from sewage, i.e., from the environment with a much higher selection pressure of antibiotic. Almost complete absence of antibiotic resistance genes in the RB2-047 genome could be explained by the isolation of the bacterial community in gold mine environment and the absence of selection pressure of antibiotic pollution.

The members of the *Acinetobacter* genus frequently possess multiple plasmids which are the key players in the adaptability of *Acinetobacter* spp. to various living conditions in the environment and in the clinical setting (Zong and Zhang 2013; Tang et al. 2020; Maslova et al. 2022). In our study, plasmid-related contigs represented just a minor fraction of the RB2-047 genome (less than 2.5%) as identified by mlplasmid server v2.1.0. We can speculate that the low number of plasmids in our isolate is the result of the spatial isolation of the gold mine environment. On the other hand, a population of 10 plasmids with genes related to the high level of tolerance to arsenic and other heavy metals were found in the environmental strain of *Acinetobacter lwoffii* obtained from a similar environment (former gold and arsenic mine in Poland) (Walter et al. 2020). Both RB2-047-1 plasmid and NODE_73 contig encode one potential TA system belonging to the type II toxin/antitoxin system RelE/ParE family toxin. Generally, RelE/ParE TA systems are linked to environmental and nutrient stress responses (LeRoux et al. 2020), which is supported by the evidence of RelE/ParE family of TA systems in plasmids of *Enterobacteriaceae* family, predominantly in *E. coli* and *Salmonella* sp., where they act as major vectors of beta-lactam, aminoglycoside and quinolone antibiotic resistance (Kamruzzaman and Iredell 2019). Although these systems were also found in various members of the genus *Acinetobacter* (Jurenaite et al. 2013; Walter et al. 2020; Alattraqchi et al. 2021), recent study appears to provide the first evidence of the RelE/ParE family in *A. johnsonii*. Nevertheless, it is not likely that such a small plasmid (≤ 5 kb) participates in the adaptation of *A. johnsonii* RB2-047 to the environmental stress.

Metal/formaldehyde-sensitive transcriptional repressor (RcnR-like protein clustered with metal resistance protein CzcD) was detected in the NODE_73 sequence. Repressor is involved in a metal tolerance in many bacteria; it mainly regulates the transcription of *rcnA* gene encoding the nickel and cobalt efflux protein (Denby et al. 2016; Mindlin et al. 2016). However, this gene was not found in the RB2-047 genome. Moreover, a metal/formaldehyde-sensitive repressor was found to regulate the IncP1-type plasmid conjugation (Zoolkefli et al. 2021).

It is accepted that metal-tolerant bacteria have a potential application in bioremediation of environments contaminated by heavy metals and organic pollutants (Ghaima et al. 2018). Two decades ago, a successful experiment was performed using *A. johnsonii* isolated from a wastewater treatment plant in UK that was able to accumulate La^3+^ from solution via precipitation of cell-bounded LaPO_4_ (Boswell et al. 2001). Bejestani et al. (2013) described the *Acinetobacter* sp. isolated from effluent samples collected in Tehran (Iran), which was not only tolerant to zinc, copper, chromium, and mercury, but also had the extensive capability to remove zinc from medium. Several other strains of *Acinetobacter* spp. have been successfully used in the bioremediation of heavy metals such as chromium, nickel, copper, zinc, lead, cadmium and uranium (Bhattacharya and Gupta 2013; Irawati et al. 2015; Li et al. 2018). *Acinetobacter* species have also been shown to be effective in oxidizing toxic As(III) to As(V) which represents a potential detoxification mechanism because it generates a less toxic and less mobile form of arsenate (Nagvenkar and Ramaiah 2009). According to recent knowledge about the multi-metal resistance and genetic features, the RB-047 strain showed a high potential for application in the bioremediation of heavy metal polluted environments, however, further investigation is needed in this area.

## Conclusion

Our study confirmed the importance of efflux activity in response to environmental stress in bacteria. We have identified several efflux pump and metal resistance genes located on the bacterial chromosome of the RB2-047 isolate. The strain showed the presence of only one chromosomally located antibiotic resistance gene bla_*OXA-211*_. Generally, environmental isolates showed significantly lower number of antibiotic resistance genes compared to the clinical isolate. This fact could lead to the hypothesis, that antibiotic tolerance in clinical bacteria is encoded mainly by specific antibiotic resistance determinants, while antibiotic and metal tolerance in environmental isolates may be the result mainly of the non-specific efflux pump activity.

### Supplementary Information

Below is the link to the electronic supplementary material.

Supplementary material 1 (PDF 413.1 kb)
