# Supplementary material for: Genomic insights into the adaptation of Acinetobacter johnsonii RB2-047 to the heavy metal-contaminated subsurface mine environment
Source: Biometals. 2023 Nov 16;37(2):371–87. doi: 10.1007/s10534-023-00555-0 (PMC11006771; doi:10.1007/s10534-023-00555-0)
Supplement: Supplementary file 1 — Supplementary material 1 (PDF 413.1 kb) [file 10534_2023_555_MOESM1_ESM.pdf]

Supplementary material

BioMetals

Genomic insights into the adaptation of *Acinetobacter johnsonii* RB2-047 to the heavy metal-contaminated subsurface mine environment

Ivana Timková[a], Lenka Maliničová[a], Lea Nosáľová[a], Mariana Kolesárová[a], Zuzana Lorková[a], Nikola Petrová[a], Peter Pristaš[a, b], Jana Kisková[a]*

[a] *Department of Microbiology, Institute of Biology and Ecology, Faculty of Science, Pavol Jozef Šafárik University in Košice, Šrobárova 2, 04154 Košice, Slovakia*

[b] *Institute of Animal Physiology, Centre of Biosciences, Slovak Academy of Sciences, Šoltésovej 4-6, 04001 Košice, Slovakia*

* corresponding author: jana.kiskova@upjs.sk

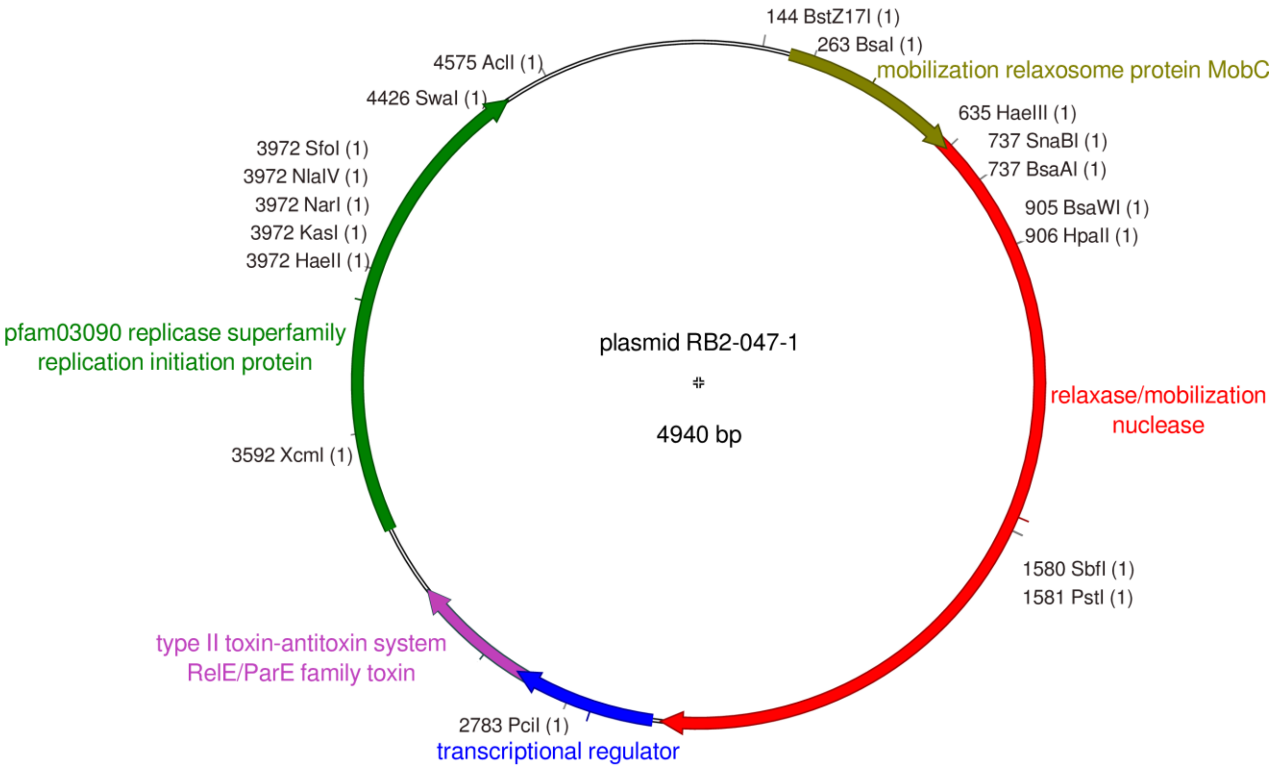

Fig. S1 Plasmid map of RB2-047-1 showing restriction enzyme sites and identified genes.

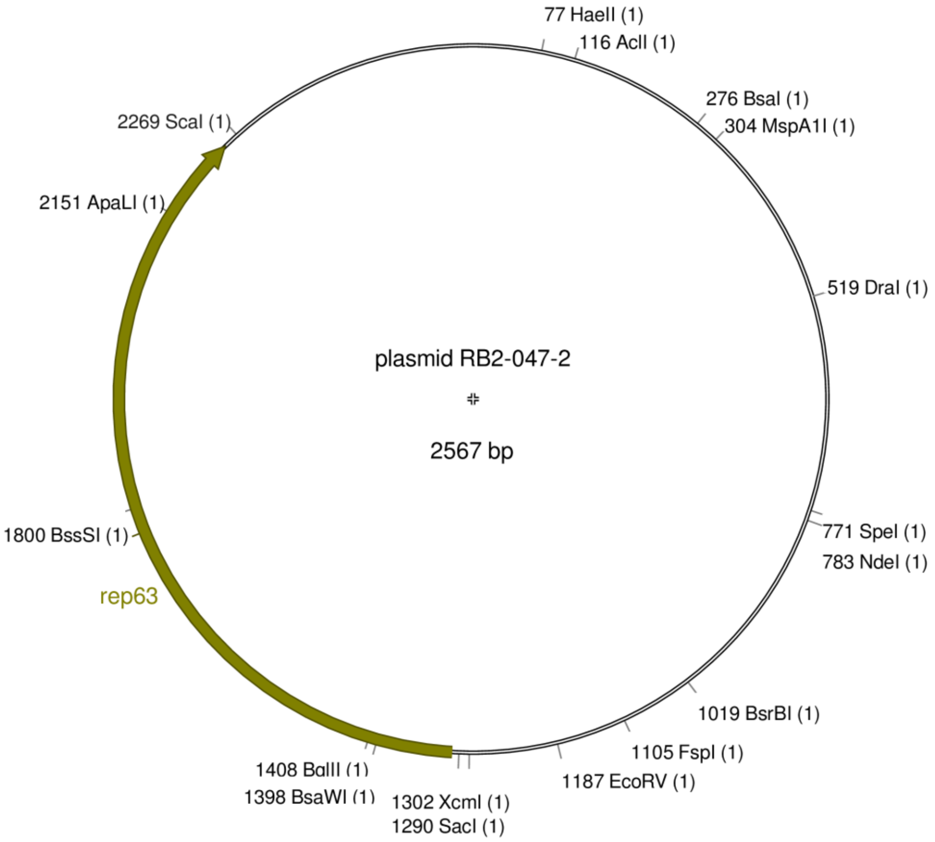

Fig. S2 Plasmid map of RB2-047-2 showing restriction enzyme sites and identified replication gene rep63.
